# Biosynthesis of glucocorticoids in tumors. Reply.

**DOI:** 10.1172/JCI175274

**Published:** 2023-11-01

**Authors:** Matthew D. Taves, Shizuka Otsuka, Jonathan D. Ashwell

**Affiliations:** Laboratory of Immune Cell Biology, Center for Cancer Research, National Cancer Institute, NIH, Bethesda, Maryland, USA.

**Keywords:** Immunology, Adaptive immunity, Cancer

**The authors reply:** In a Letter to the Editor regarding our recent paper (1), Cirillo (2) challenged our conclusion that tumors produce glucocorticoids primarily via 11β-HSD1–mediated metabolite recycling rather than Cyp11b1-mediated de novo synthesis. Our responses to the main points follow.

*De novo glucocorticoid synthesis by tumors is well-established.* Although there is a literature showing tumor glucocorticoid production, the evidence that glucocorticoids are primarily synthesized de novo and in sufficient amounts to affect tumor immunity is not “solid.” In the review cited by Cirillo (3), we found three studies that directly demonstrated glucocorticoid synthesis by precursor (progesterone or 11-deoxycorticosterone [DOC]) conversion to glucocorticoids. In two studies this was quantified, and precursor conversion by human melanoma cell lines was negligible (<1%). This parallels our results with mouse melanoma cell lines. In contrast with Cyp11b1-mediated de novo synthesis, 11β-HSD1 efficiently converted dehydrocorticosterone (DHC) to glucocorticoids (100%). Most other studies examining extraadrenal glucocorticoid production (3) inferred that the glucocorticoids were synthesized de novo because production was prevented by the Cyp11b1 inhibitor metyrapone. However, metyrapone also inhibits 11β-HSD1 and cannot discriminate between synthesis and regeneration. Furthermore, even if de novo synthesis is detectable, without side-by-side comparison of Cy11b1 versus 11β-HSD1 activity their relative importance is unknown. We are unaware of such comparisons of tumor cells prior to our study, but in normal mouse skin (4) corticosterone production by 11β-HSD1 dwarfed that of Cyp11b1, as we observed.

*Species differences might lead to misleading conclusions regarding human cancers.* We agree that the mechanism of glucocorticoid production is important, and, therefore, we analyzed *CYP11B1* (synthesis) and *HSD11B1* (regeneration) expression in human cancers. *HSD11B1* but not *CYP11B1* was upregulated in multiple cancers and was widely and strongly associated with expression of glucocorticoid-responsive genes, regulatory T cell markers, and effector T cell exhaustion markers. This paralleled our findings in mice and strongly supports the notion that 11β-HSD1 (not Cyp11b1) produces biologically significant levels of cortisol in these human tumors, with a negative clinical impact. Our findings have been mirrored by those of another group independently converging on 11β-HSD1 as a target for cancer therapy, using mouse models and human cancer data sets (5).

*De novo–synthesized corticosterone might have been produced but then converted to aldosterone by Cyp11b2.* Cirillo supposes that the tumor cells we analyzed did synthesize corticosterone via Cyp11b1, but that it was all converted to aldosterone by coexpressed Cyp11b2. If this were true, the synthesized corticosterone would simply be an intermediary and transient metabolite, not a secreted bioactive product. More importantly, 11β-HSD1–generated corticosterone from the same cells would *also* have been depleted by conversion to aldosterone. This point exposes a self-contradiction in this model: efficient corticosterone conversion to aldosterone would preclude its detection from all sources. Nonetheless, we have tested this possibility and found that *Cyp11b2* expression was extremely low in B16.F10 (B16) melanoma and MC38 colorectal tumor cells (Figure 1A). Although both converted DHC to corticosterone, neither converted DOC to corticosterone or aldosterone (Figure 1B), ruling out a role of Cyp11b2.

*Our study does not contradict previous evidence of tumor synthesis of glucocorticoids.* We did not claim that tumors can never synthesize biologically significant levels of glucocorticoids, and some certainly do (this clearly occurs with adrenal adenomas and, perhaps, murine AOM-DSS–induced colorectal tumors, ref. 6). However, for the seven glucocorticoid-producing tumor cells we examined, the amounts regenerated by 11β-HSD1 were vastly greater than those synthesized by Cyp11b1, and inhibiting 11β-HSD1 substantially enhanced antitumor immunity and reduced tumor growth. In humans, *HSD11B1* was expressed in many cancer types, but *CYP11B1* and *CYP11B2* had low or absent expression (Figure 1C). The data thus far support the notion that 11β-HSD1–mediated regeneration, not Cyp11b1-mediated synthesis, is the major source of biologically, and perhaps clinically, relevant tumor-derived glucocorticoids.

*Data availability.* Values for all data points in graphs are reported in the Supporting Data Values file.

## Supplementary Material

Supporting data values

## Figures and Tables

**Figure 1:**
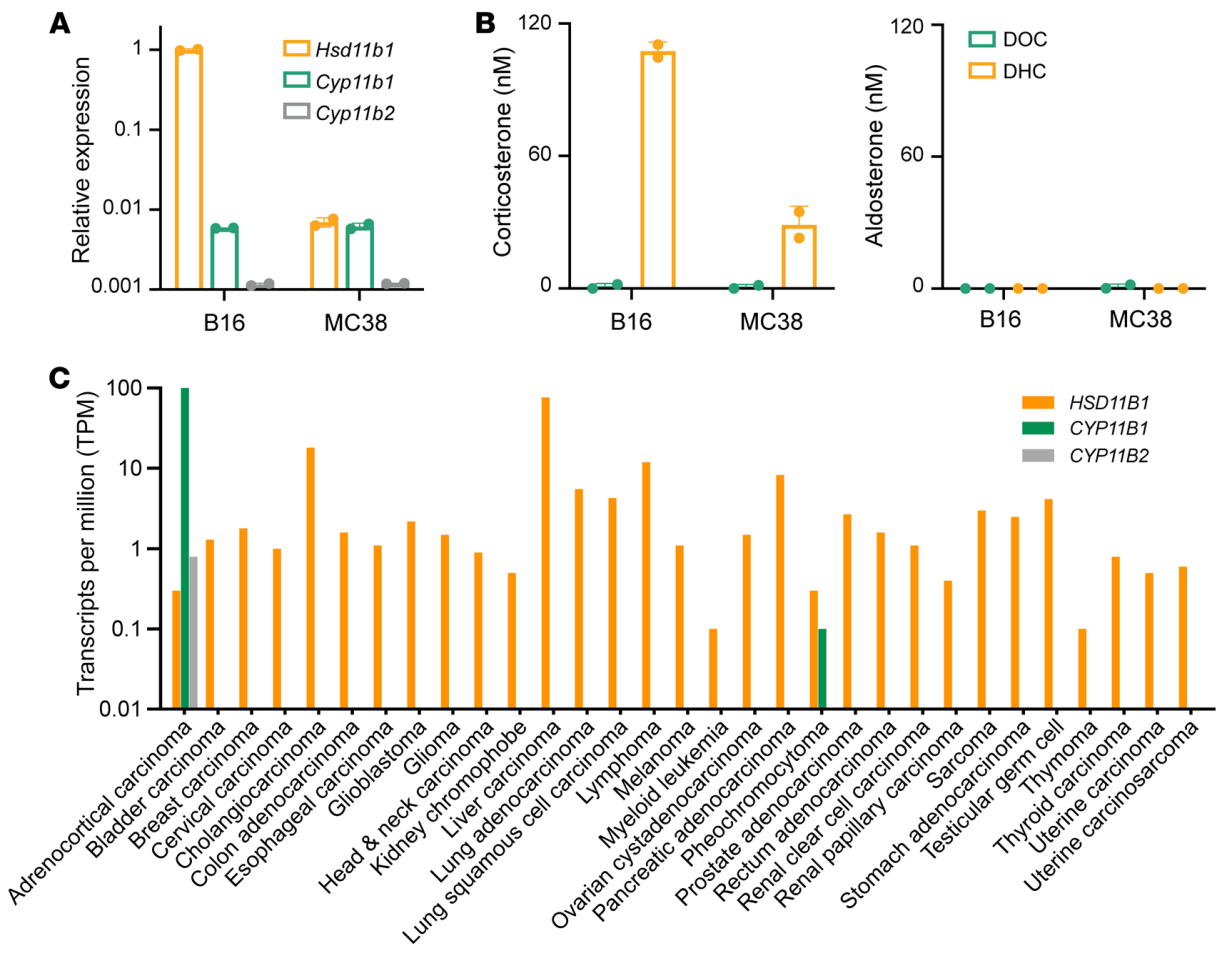
Glucocorticoid-producing enzyme expression in tumors. (**A**) Relative *Hsd11b1*, *Cyp11b1*, and *Cyp11b2* expression (corrected for *Gapdh*) in tumor cells was determined by reverse-transcription qPCR. (**B**) 2.5 × 10^4^ tumor cells were cultured with 100 nM DOC or DHC for 24 hours, and steroids were assayed by ELISAs (Arbor Assays). (**C**) Relative gene expression in human cancers. TCGA gene expression data were analyzed using Gene Expression Profiling Interactive Analysis (http://gepia.cancer-pku.cn/), and median values are shown.

## Version 1 11/01/2023

Electronic publication
